# Identification of a New *Alcaligenes faecalis* Strain MOR02 and Assessment of Its Toxicity and Pathogenicity to Insects

**DOI:** 10.1155/2015/570243

**Published:** 2015-01-18

**Authors:** Rosa Estela Quiroz-Castañeda, Ared Mendoza-Mejía, Verónica Obregón-Barboza, Fernando Martínez-Ocampo, Armando Hernández-Mendoza, Felipe Martínez-Garduño, Gabriel Guillén-Solís, Federico Sánchez-Rodríguez, Guadalupe Peña-Chora, Laura Ortíz-Hernández, Paul Gaytán-Colín, Edgar Dantán-González

**Affiliations:** ^1^Centro de Investigación en Biotecnología, Universidad Autónoma del Estado de Morelos, 62210 Cuernavaca, MOR, Mexico; ^2^Facultad de Ciencias, Universidad Autónoma del Estado de Morelos, 62210 Cuernavaca, MOR, Mexico; ^3^Instituto de Biotecnología, Universidad Nacional Autónoma de México, 62210 Cuernavaca, MOR, Mexico; ^4^Centro de Investigaciones Biológicas, Universidad Autónoma del Estado de Morelos, 62210 Cuernavaca, MOR, Mexico

## Abstract

We report the isolation of a bacterium from *Galleria mellonella* larva and its identification using genome sequencing and phylogenomic analysis. This bacterium was named *Alcaligenes faecalis* strain MOR02. Microscopic analyses revealed that the bacteria are located in the esophagus and intestine of the nematodes *Steinernema feltiae, S. carpocapsae*, and *H. bacteriophora*. Using *G. mellonella* larvae as a model, when the larvae were injected with 24,000 CFU in their hemocoel, more than 96% mortality was achieved after 24 h. Additionally, toxicity assays determined that 1 *μ*g of supernatant extract from *A. faecalis* MOR02 killed more than 70% *G. mellonella* larvae 96 h after injection. A correlation of experimental data with sequence genome analyses was also performed. We discovered genes that encode proteins and enzymes that are related to pathogenicity, toxicity, and host/environment interactions that may be responsible for the observed phenotypic characteristics. Our data demonstrates that the bacteria are able to use different strategies to colonize nematodes and kill insects to their own benefit. However, there remains an extensive group of unidentified microorganisms that could be participating in the infection process. Additionally, a nematode-bacterium association could be established probably as a strategy of dispersion and colonization.

## 1. Introduction 

Nematodes have a global impact on ecosystems and economies; however, parasitic nematodes also can have many beneficial impacts on human interests and health [1]. For example, entomopathogenic nematodes (EPNs) that belong to the families Steinernematidae and Heterorhabditidae are commercially used as biological control agents for crop pests [2]. The EPNs* Heterorhabditis* and* Steinernema* have unique mechanisms to associate with and transmit bacteria to insect hosts; specifically, these nematodes have a symbiotic association with pathogenic bacteria from the* Photorhabdus* and* Xenorhabdus* genera, respectively [3]. In this relationship, the nematode provides the bacteria with nutrients, protection, and environmental dispersion. Meanwhile, the bacteria provide nutrition to the nematode through available insect-derived nutrients as well as antimicrobial compounds that prevent the development of bacteria, fungi, and yeast in the insect. For example,* Photorhabdus* produces bacteriocins and lumicins [4, 5].

Both nematodes and bacteria can infect and kill insects. Once the infective juvenile reaches the insect hemocoel, they release the symbiotic bacteria into a rich medium that grows the bacterial cells. The bacterial cells then release antimicrobial compounds, toxins, and exoenzymes causing the insect death usually within 24–48 h [6, 7].

Although it has been postulated that the nematode-bacteria interaction is unique some nonsymbiotic bacteria that are able to coinhabit or colonize the insect cadaver and the nematode have been reported. Lysenko and Weiser [8] isolated bacteria associated with* S. carpocapsae*, such as* Alcaligenes, Pseudomonas*, and* Acinetobacter* spp., which also are pathogenic to* Galleria mellonella* larvae. More recently, the bacteria* Flavobacterium* sp.,* Providencia vermicola*, and* Alcaligenes faecalis* were isolated from the nematode* Rhabditis blumi* [9].

Several bacterial species have also been identified from hemolymph of insect cadavers infected with EPNs as well as from infective juvenile EPNs [4].

In this study, we report the isolation and identification of the bacteria* A. faecalis* strain MOR02 from* G. mellonella* dead larva recovered from soil samples in Tenango, Morelos, Mexico. The pathogenicity and toxicity of this bacterium were also analyzed and we determined that* A. faecalis* MOR02 causes mortality in* G. mellonella *larvae 24 h after injection. Additionally, the protein extract from a supernatant culture of the bacteria is toxic in* G. mellonella* larvae 96 h after injection. We also microscopically observed the association of* A. faecalis* MOR02 and nematodes from the genera* Steinernema *and* Heterorhabditis. *We performed genome sequencing on* A. faecalis* MOR02, and the bioinformatic data analysis supports the phenotypic characteristics of the bacteria.

## 2. Materials and Methods 

### 2.1. Bacterial Isolation from the Nematode

The bacteria were isolated from the hemolymph of a* G. mellonella* larvae cadaver found in the soil of Tenango (Santa Ana), Morelos, Mexico, by Guadalupe Peña.* G. mellonella* larvae were disinfected with ethanol 70% and the contents were streaked on LB media plates for bacterial growth. The isolated bacteria were grown at 30°C at 250 rpm overnight and then were grown on LB media plates.

Bacterial genomic DNA extraction was performed using the UltraClean Microbial DNA Isolation kit (MOBIO).

### 2.2. Bioinformatic Methods

#### 2.2.1. Assembly

For the sequencing of the* A. faecalis* MOR02 genome, sequencing on the Genome Analyzer IIx (GAIIx) Illumina platform was performed by the UUSMD (Unidad Universitaria de Secuenciación Masiva de DNA, Instituto de Biotecnología, UNAM). The 19,250,362 paired-end reads were assembled de novo using the SPAdes program (version 3.1.1) and 23 contigs were generated with a 315-fold median coverage depth.

#### 2.2.2. Annotation

The RAST (Rapid Annotation using Subsystem Technology) version 2.0 (http://rast.nmpdr.org/) [10], RNAmmer version 1.2 (http://www.cbs.dtu.dk/services/RNAmmer/) [11], tRNAscan-SE version 1.21 (http://lowelab.ucsc.edu/tRNAscan-SE/) [12], and ARAGORN (http://mbioserv2.mbioekol.lu.se/ARAGORN/) [13] servers were used for genome annotation and the prediction of rRNA and tRNA genes, respectively. Clusters of Orthologous Groups of proteins (COG) [14] and Gene Ontology (GO) [15, 16] annotations were performed using a BLAST search against the downloaded databases. KEGG Orthology (KO) [17, 18] annotation was performed at the KEGG Automatic Annotation Server (KAAS) with the Bidirectional Best Hits (BBH) method (http://www.genome.jp/kegg/kaas/). PFAM annotation was performed at the EMBL-EBI batch search server (http://pfam.xfam.org/search).

#### 2.2.3. Phylogeny and Identification

The isolated 16S rRNA sequence was identified using the RNAmmer version 1.2 server [11] (http://www.cbs.dtu.dk/services/RNAmmer/) on contig1 (722,086 bp). This sequence was used for a BLASTN search (http://blast.ncbi.nlm.nih.gov/) using the MEGABLAST algorithm to search for nonredundant (nr) nucleotide [19] databases. The higher significant alignments reported were downloaded for phylogenetic analysis. A full tree with 165 sequences and a representative tree with 25 sequences of the genera* Alcaligenes* and closely related bacteria were constructed using all 16S rRNA sequences. These sequences were first aligned using MUSCLE server version 3.7 (http://phylogeny.lirmm.fr/phylo_cgi/one_task.cgi?task_type=muscle) [20] and the resulting alignment was analyzed using the phylogenetic analysis program MEGA version 6.0 [21]. A maximum likelihood method was used to calculate distances and the neighbor-joining method was used to infer the evolutionary tree and bootstrap values were also calculated using 1000 replicates.

#### 2.2.4. Phenotypic Characterization

Three tests were performed as part of phenotypic characterization: the swarming motility test, chitinase activity assay, and esterase/lipase hydrolysis test.

To assess swarming motility, we slightly modified a previously reported method [22]. Briefly, a plate of trypticase soy broth (TSB) (Bioxon) and 0.5% agar (Bioxon) was inoculated in the center with a drop of a bacteria enriched liquid culture that was incubated at 25°C for 7 days.* E. coli* DH5*α* that have reportedly poor motility were used as a control [23].

For the chitinase activity assay, a sterile solution containing colloidal chitin was prepared as follows: 2 g colloidal chitin was dissolved in 100 mL of a solution prepared containing 0.5 g casein peptone (Bioxon), 0.5 g yeast extract (Bioxon), 0.1 g KH_2_PO_4_ (High Purity) 0.01 g MgSO_4_ *·*7H_2_O (J.T. Baker), 1.5 g agar (Bioxon), and pH adjusted to 6.5. A drop of an enriched culture of* A. faecalis *MOR02 was inoculated on the plates and incubated at 37°C until a white halo of degradation was observed in the center of the plate.* E. coli* DH5*α* cells were used as a negative control. The streptococcus thermophiles activity of the bacteria were assessed using the Tween 80 hydrolysis test that was performed as previously reported [24].

#### 2.2.5. Cell Transformation

Electrocompetent* A. faecalis* MOR02 cells were prepared and transformed using electroporation with the plasmid pT4-23S-Cherry. This plasmid has the coding sequence of mCherry under the control of a 23S promoter and a kanamycin resistance cassette (30 *μ*g/mL). We have previously constructed this plasmid based on the pT4-mCherry plasmid, which has coding sequence of mCherry under the control of the* trc *promoter and a kanamycin resistance cassette (30 *μ*g/mL). After 3 days of growth at 37°C, positive red clones were observed and fluorescence microscopic observation was performed using a filter Nikon B-2A (Nikon Eclipse E4000). We subsequently referred to this transform as* A. faecalis* MOR02-Cherry.

#### 2.2.6. Bacterial Pathogenic Assays

For all pathogenic assays, 100 mL of LB medium (kanamycin 100 mg/mL) was inoculated with a fresh preinoculum of* A. faecalis* MOR02-Cherry; the cells were then grown at 37°C overnight. When an OD_600_ of 1.4–1.6 was reached, the cells were collected using centrifugation at 5,000 g at 4°C for 20 min. Serial dilutions were performed in LB broth to obtain dilutions of 10^8^, 10^9^, 10^10^, and 10^11^, which corresponded to bacterial suspensions at 2.4 × 10^4^–2.4 × 10^7^ CFU/mL. The same conditions were used for negative control* E. coli* DH5*α* transformed with pT4-mCherry, a plasmid with coding sequence of mCherry under the control of the* trc *promoter and a kanamycin resistance cassette (30 *μ*g/mL).

The pathogenicity assessment was performed using the injection method on sixth-instar* G. mellonella* larvae. For injection assays, 10 *μ*L of bacterial suspensions was injected into the dorsal region of the third from last abdominal segment of the larvae using a 0.3 mL/cc insulin syringe. Before each pathogenic assay, the presence of* A. faecalis* MOR02-Cherry in cultures used to inject the* G. mellonella* larvae was verified using fluorescence microscopy (Nikon Eclipse E4000).

LB broth and* E. coli* DH5*α* cells carrying the pT4-mCherry plasmid were used as a negative control. Additionally, damage caused by the puncture was also assessed in the larvae. Each bacterial suspension was tested using 10 insect larvae in three independent experiments.

The injected larvae were incubated at 26°C in presence of small pieces of diet [for 500 mg of diet, 97.5 mL bee honey, 20 mL sterile glycerol (J.T. Baker), 37.5 g sterile wheat bran, 73 g sterile rice cereal, and 73 g yeast extract (Pronat Ultra)]. Mortality was assessed every 24 h after injection. We considered dead or dying larvae when they did not show any movement after being pricked with a toothpick as well as a black appearance due to necrosis.

At 24 and 48 h after injection the inner contents of the* G. mellonella* larvae were streaked on LB media Petri dishes supplemented with kanamycin 100 mg/mL and grown at 37°C for 48 h.* A. faecalis* MOR02-Cherry cells were identified by the presence of red fluorescence. In each case, three independent experiments were performed.

#### 2.2.7. Protein Precipitation and Toxicity Assay

The proteins present in 300 mL of supernatant from* A. faecalis* MOR02-Cherry cultures were precipitated using cold acetone and were incubated at −20°C overnight. The acetone cultures were centrifuged at 5,000 g at 4°C and the protein pellet was obtained and washed twice with cold acetone. After the acetone evaporated, the pellet was resuspended in Tris 10 mM pH 7.2. Proteins were quantified using the Bradford reagent (BioRad) and a standard curve of bovine serum albumin (BSA).

To assess protein toxicity, sixth-instar* G. mellonella* larvae were injected in the hemocoel with 0.5 , 1, 2, and 4 *μ*g extracted proteins (final volume injected in each larvae, 20 *μ*L). The negative controls were larvae injected with BSA at the same protein concentration and Tris 10 mM pH 7.2. We also used larvae that were only punctured with the syringe to evaluate damage due to the injection. For each protein concentration, 10 larvae were injected and three independent experiments were performed.

#### 2.2.8. Association of* A. faecalis* Strain MOR02 with Nematodes

To establish whether an association between the nematode and bacteria existed, second stage infective juvenile* S. feltiae, S. carpocapsae,* and* H. bacteriophora* nematodes (Entonem, Koppert) were hydrated using sterilized distilled water and then were put in contact with* A. faecalis *MOR02-Cherry. The bacteria cells were previously grown on LB media dishes supplemented with kanamycin (100 mg/mL) for 48 h at 37°C.

In 60 × 15 mm Petri dishes, the nematodes (approximately fifty) and a loopful of bacteria were mixed and incubated at 20°C for 18 h. After this, the mCherry protein in the nematodes was excited at 555 nm and emission fluorescence was collected at 605 nm using a bandpass of 40X (Nikon, TE300).

### 2.3. Statistical Analysis

All statistical analyses were performed using Minitab 15 Statistical Software. A one-way analysis of variance [25] was used to analyze differences in mortality when different amounts of supernatant extract were used in the bioassays (1, 2, and 4 *μ*g). The same analysis was performed to assess differences in mortality among different injected CFU numbers in the larvae. To determine significant differences among the means, a Tukey test was performed. The significance threshold was set at *P* < 0.05.

## 3. Results

### 3.1. Bacterial Isolation

After the bacteria were isolated from the larva, they were grown in liquid LB medium and then were streaked on Petri dishes with solid LB medium. From these isolates, one colony was selected and used for further genomic DNA extraction and identification.

### 3.2. Genome Sequencing

The genome of the isolated microorganism was sequenced using the Genome Analyzer IIx (GAIIx) Illumina platform, with a random subset of 19,250,362 paired-end reads (315X coverage). The genome was assembled using the SPAdes program (version 3.1.1) into 23 contigs that were deposited in the GenBank database. We obtained a draft genome with a total length of 4,402,705 bp in the 23 contigs that had a GC content of 56.4% [26].

### 3.3. Identification

The genome draft was analyzed on the RAST server (version 2.0) [10] and contains 4,019 coding sequences (CDS) including 52 tRNAs. Using the servers RAST, ARAGORN [27] (http://mbio-serv2.mbioekol.lu.se/ARAGORN/), and RNAmmer 1.2 [11] (http://www.cbs.dtu.dk/services/RNAmmer/) to analyze the contig1 sequence (722,086 bp) we identified the presence of the genes coding for tRNA-Val(gac) [121,713-121,789], tRNA-Leu(tag) [324,903-324,987] and tRNA-Met(cat) [456,223-456,301], in addition to 5S rRNA [609,890-610,001], 23S rRNA [610,185-613,067] tRNA-Ala(tgc) [613,447-613,522], tRNA-Ile(gat) [613,534-613,610], and 16S rRNA [613,709-615,233] in that order (Figure S1 in Supplementary Material available online at http://dx.doi.org/10.1155/2015/570243). This arrangement is closely similar to the one reported for an* A. faecalis* subsp.* faecalis* NCIB 8687 contig (GenBank accession number AKMR01000044; 6,924 bp), but without the presence of the tRNA-Val-GAC gene described in that strain. The sequence of the 16S rRNA gene (1,524 bp) reported here is 99% identical to several* A. faecalis* strains that are reported in the GenBank database. These strains are described as being involved with (1) converting iminodiacetonitrile to iminodiacetic acid, (2) phenol degradation, (3) host of* S. thermophilus*, or (4) synthesis of (R)-(-)-mandelic acid and its derivatives from racemates by enantioselective degradation, among other activities. However, these isolated strains have not been completely characterized or published.

**Figure 1:**
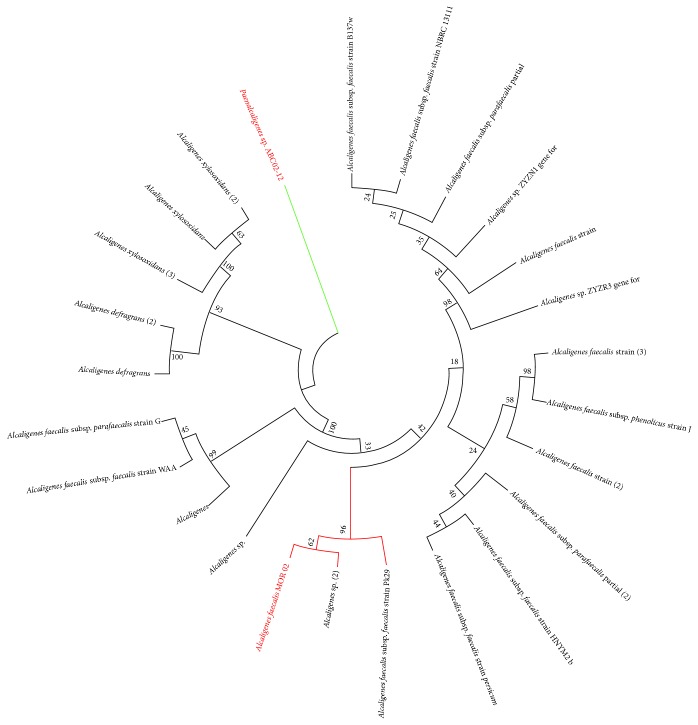
16S rRNA gene phylogeny of 24 Alcaligenes spp., including* A. defragrans*,* A. xylosoxidans*, and several substrains of* A. faecalis*. In addition, a related bacterium isolated from larvae guts,* Paenalcaligenes* sp. ABC02-12, was used as an outgroup (green line). Maximum likelihood method was used to compute the evolutionary distances and the neighbor-joining method was used to infer the evolutionary history. Bootstrap percentages are given in the nodes (number of bootstrap replicates: 1000).* A. faecalis* strain MOR_02 is shown in red.

Based on the results of the 16S ribosomal DNA (rDNA) sequence (Figures 1 and S2), this strain was the closest to (i)* A. faecalis *BC2000 (GenBank accession number AY662683.1; homology 99%, based on 16S rDNA), which is one of a group of bacteria isolated from rhizosphere soil. BC2000 has been proven to have functions on plant growth-promotion and antagonism against plant parasitic nematodes; (ii)* Alcaligenes* sp. F78 (GenBank accession number EU443097.1; homology 99%, based on 16S rDNA), isolated from a mycorrhizosphere bacteria conglomerate; (iii)* Alcaligenes* sp. ECU0401 (GenBank accession number EF535732.1; homology 99%, based on 16S rDNA), a nitrilase producer; and (iv)* Alcaligenes* sp. PGBS001 (GenBank accession number EU622578.1; homology 99%, based on 16S rDNA) isolated from a microbial community decomposing wheat straw under aerobic conditions (Figure S2).

We conclude that our strain is an* A. faecalis* and designated it as* A. faecalis* MOR02 (GenBank accession number JQCV00000000, http://www.ncbi.nlm.nih.gov/nuccore/JQCV00000000.1/).

### 3.4. Genome Annotation

Our draft genome provided us with information concerning the potential proteins involved with several of the observed phenotypes. The 4,019 CDS reported by the RAST server (version 2.0) were compared with the Clusters of Orthologous Groups of proteins (COG), Gene Ontology (GO) [15], KEGG Orthology [17], and PFAM databases. After the predicted proteins were classified based on these different approaches, we use several keywords (e.g., symbiosis, pathology, intracellular survival, toxicity, and antibiotic production) to cluster the candidates with similar functions. Finally, the candidate proteins were grouped and are summarized in Figure 5.

The most abundant proteins found in our search were those involved with pathogenesis and toxicity, followed by drug/antibiotic resistance proteins.  Table 2 shows the nonredundant (nr) proteins retrieved from RAST server (version 2.0) and analyzed databases. The host/environment interactions subgroup includes proteins related to flagellar movement and taxis, which could participate in the observed* A. faecalis* MOR02 swarming movement. The proteins related to both activities are chemotaxis proteins, chemotaxis response protein, flagellin, flagellar transcriptional activators, flagellar biosynthetic proteins, flagellar basal-body rod proteins, and flagellum specific ATP synthase, among others (Table S1).

The subgroup pathogenicity and toxicity primarily include peptidases, proteases, lipases/esterases, hemolysins, virulence factors, and toxins. The chitinase activity of* A. faecalis* MOR02 may be related to a chitin deacetylase protein found in the COG database, whereas the activity on Tween 80 may be due to the presence of lipases/esterases. As we reported, the supernatant extract of* A. faecalis* MOR02 was toxic to* G. mellonella *larvae; this toxicity must be due to proteins that are excreted, specifically toxins, proteases, and peptidases. In the bacteria genome we found some genes that encode proteins that could be participating in such toxic activities. In the GO database, we found that there is evidence that the TolR protein acts as a toxin transporter and a toxin secretion ATP-binding protein; therefore, it may participate in pathogenesis. We also found proteins in the type II secretion pathway, a system that Gram-negative bacteria use to release enzymes or toxins (Tables S2, S3, and S4). Finally, in the antibiotic synthesis and drug/antibiotic resistance subgroups, we found genes that encode proteins related to antibiotic synthesis and resistance, such as proteins involved in mitomycin antibiotic biosynthesis, ABC-type bacteriocin/antibiotic exporters, beta-lactamases, MATE (multidrug and toxic compound extrusion) efflux family proteins, and multidrug resistance transporters. We also found other proteins that were more specific, such as chloramphenicol acetyltransferases or translation elongation factor G that has a known function in tetracycline resistance, among others. We also found additional proteins related to drug resistance such as bacitracin, bleomycin, and fusaric acid in PFAM database (Tables S1, S3, and S5). All of these proteins were reported to participate in the mechanisms that bacteria possess to cope with antibiotics.

### 3.5. Phenotypic Characterization

Extracellular chitinase production was discovered when we observed a white halo of chitinase activity after incubating* A. faecalis* MOR02 with chitin. Esterase activity was observed when* A. faecalis* MOR02 used Tween 80 as a substrate and fatty acids were released. These results confirm the chitinolytic and esterase activity of* A. faecalis *that has previously been reported [28, 29]. Swarming motility was observed in TSA (Trypticase Soy Agar) plates inoculated with the bacteria, where zones of consolidation or terraces, commonly known as bull's eye, were observed. Concurrent with this observation we also observed a greenish slime surrounding the bacterial growth.

### 3.6. mCherry Protein Expression in* A. faecalis* MOR02

To analyze the possible association of* A. faecalis* MOR02 with nematodes we transformed bacteria with plasmid pT4-23S-mCherry. The transformed bacteria were referred to as* A. faecalis* MOR02-Cherry and mCherry expression was corroborated using fluorescence microscopy.

### 3.7. Pathogenicity of* A. faecalis* MOR02 to* G. mellonella *Larvae

To assess the ability of* A. faecalis* MOR02 to grow and survive within the hemocoel larvae as well as to test its pathogenicity, 10 *μ*L of different bacterial suspensions of* A. faecalis* MOR02-Cherry was injected intolarvae. As shown in Table 1, larvae injected with 240 and 2,400 CFU had 3.33% mortality at 72 h after injection and 6.67% at 48 h after injection, respectively. With 2,400 CFU, only 33.33% mortality was observed at >96 h after injection. Conversely, larvae injected with 24,000 CFU had 96.67% mortality at 24 h after injection, while 100% mortality was observed using 240,000 CFU.

We did not observe any mortality as a result of injection puncture, injection of LB broth or* E. coli* DH5*α* cells.

### 3.8. Toxicity of the Supernatant Protein Extract of* A. faecalis* MOR02

**Figure 2:**
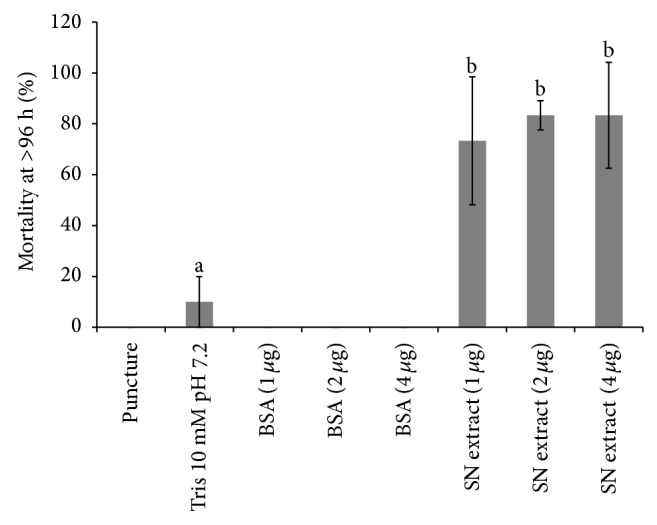
Toxicity of the supernatant (SN) extract of* A. faecalis *MOR02 on* G. mellonella* larvae. At 96 h after injection, 73.3, 83.3, and 83.3% of larvae were dead using 1, 2, and 4 *μ*g toxin extract, respectively. There were no significant differences between the treatment groups. BSA: bovine serum albumin; puncture: larvae only punctured with a syringe. Different letters indicate significant differences. Bars represent standard deviation of three independent experiments.

A toxicity comparison of four different amounts of supernatant protein extracts was assayed using sixth-instar* G. mellonella* larvae as the host. All doses had toxic activity as indicated by the presence of dead larvae at 96 h after injection. Necrotic tissue was also observed in the insects, as well as a change in body size and consistency. At 96 h after injection, 73.3, 83.3, and 83.3% of the larvae were dead using 1, 2, and 4 *μ*g protein extract, respectively. Mortality was not observed in larvae that were punctured or injected with BSA (1, 2, and 4 *μ*g) and the Tris 10 mM pH 7.2 controls resulted in 10% mortality (Figure 2).

### 3.9. The Association of* A. faecalis* Strain MOR02 with Nematodes

**Figure 3:**
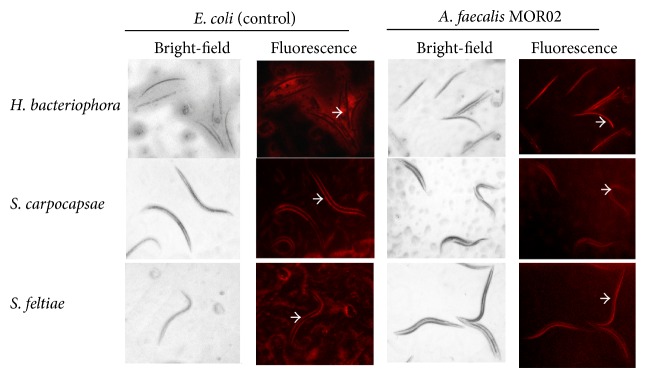
Association of* A. faecalis* MOR02 IJ2 nematodes. Bright-field and fluorescence microscopy analyses of the association of* E. coli* and* A. faecalis *MOR02-Cherry with* H. bacteriophora, S. carpocapsae*, and* S. feltiae. *In* E. coli* (negative control), fluorescence is observed outside nematodes (white arrows), whereas* A. faecalis* MOR02 fluorescence is located inside the nematodes (white arrows). Gut autofluorescence of* H. bacteriophora* is not clearly observed due to red fluorescence of* A. faecalis* MOR02.

**Figure 4:**
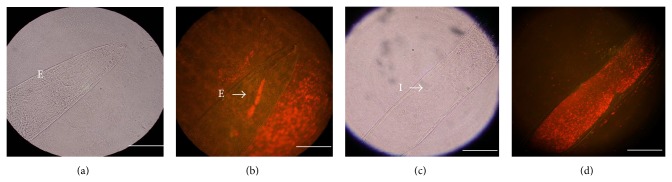
Microscopic analysis of the association of* A. faecalis *MOR02 with IJ2* S. feltiae*. (a) Bright-field microscopy of the anterior section of the nematode; E, esophagus. (b) Fluorescence microscopy of the anterior section of the nematode showing the bacteria located in the nematode esophagus; the arrow points to* A. faecalis *MOR02-Cherry inside the nematode esophagus; (c) Bright-field microscopy of the nematode intestine; I, intestine. (d) Fluorescence microscopy of the intestine with bacterial colonization. Scale bar, 25 *μ*m.

**Figure 5:**
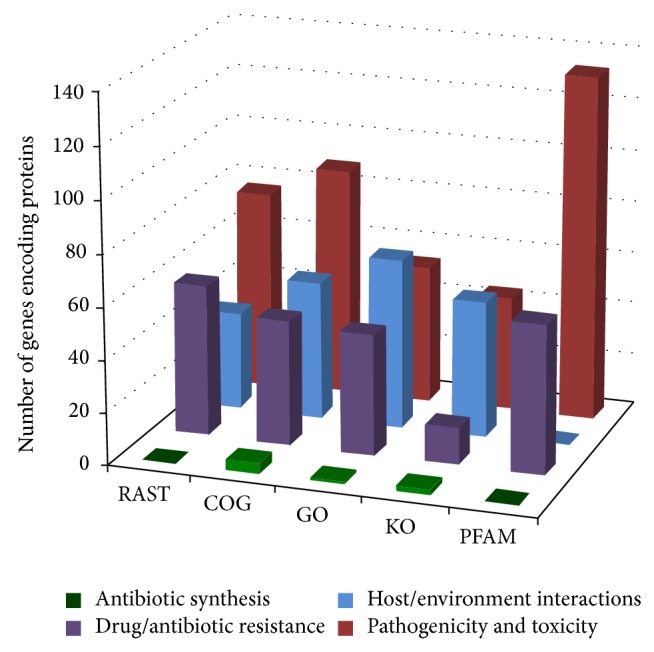
The number of genes encoding proteins and their classification according to information retrieved from the RAST server and COG, GO, KO, and PFAM databases.

A microscopic analysis of the nematodes showed that* A. faecalis* MOR02-Cherry is located inside the nematodes* S. feltiae, S. carpocapsae*,and* H. bacteriophora *after 18 h at 20°C. We also observed fluorescence outside of the nematodes in the presence of* Escherichia coli *expressing mCherry protein (negative control) (Figure 3). The fluorescence in* S. feltiae *nematodes was observed along the esophagus and intestine (Figure 4).

## 4. Discussion

We recently identified a bacterium that we named* A. faecalis* MOR02 that was isolated from a nematode found in a* G. mellonella* larva cadaver.

The chitinase and esterase activity that were observed as part of the phenotypic characterization of* A. faecalis* MOR02 are supported by the presence of genes related to these activities within its genome. One such gene is chitin deacetylase that has been reported in fungi and insects, but only in the bacteria* Bacillus *sp.,* Serratia *sp., and members of the family Vibrionaceae [30–32]. This enzyme catalyzes the deacetylation of chitin to produce acetate and chitosan, which indicates that this enzyme has potential biotechnological applications for controlling fungal plant pathogens or insect pests in agriculture. Both chitinase and esterase activities may assist the bacteria when degrading insect body components, which we observed as a floppy phenotype (massive loss of body turgor) in* G. mellonella* larvae infected with* A. faecalis *MOR02.

The swarming motility by flagella in* A. faecalis* MOR02 has been reported in this species as well as in alphaproteobacteria, gammaproteobacteria, and firmicutes. Actually, this behavior is considered a pathogenic factor for eubacteria, such as* Proteus mirabilis*,* Pseudomonas aeruginosa, Serratia liquefaciens*, and* S. marcescens* [33–35]. Several proteins, such as flagellin and those related to flagellar body composition, were also identified in the databases we analyzed.

The diverse associations in nematodes are of interest because they represent examples of diversity, adaptation, and evolution in nature. When we examined the association between* A. faecalis* MOR02 and the nematodes* S. feltiae*,* S. carpocapsae*, and* H. bacteriophora*, we observed that fluorescence clearly shows that* A. faecalis* MOR02 is located in the digestive tract of nematodes, whereas the control* E. coli* was observed outside on the cuticles of nematodes. This result demonstrates the specificity of the bacteria-nematode interaction. The results of the pathogenicity assays indicated that* A. faecalis *MOR02 is lethal when directly injected into the hemocoel of larvae. However, the controls LB broth and* E. coli* DH5*α* did not affect insect mortality or changes in body consistency or development. The pathogenicity of* A. faecalis *in insects has been previously reported; Park et al. [9] observed less than 30% mortality in fourth-instar* G. mellonella* larvae at 48 h after haemocoelic injection with 10^3^, 10^4^, and 10^5^ bacteria cells of* A. faecalis.* In this study, we observed 96% mortality 24 h after injection of 24,000 CFU in sixth-instar larvae. All the dead larvae had necrosis and a softened consistency, which suggests that* A. faecalis* MOR02 enter the hemocoel and disperse throughout the insect body, excreting toxic compounds that cause the observed damage. In the* A. faecalis* MOR02 genome we found many genes that encode proteins in the type II secretion system that is conserved in Gram-negative bacteria. The proteins secreted by this pathway include proteases, pectinases, phospholipases, lipases, and toxins. Many of these proteins are associated with tissue destruction, which contributes to cell damage and disease [36, 37].

It is necessary to identify the compounds that cause toxicity after the bacteria enter the insect larvae. Genes that encode proteases and peptidases have been identified in the* A. faecalis *MOR02 genome. However, when we examined the bibliography for other candidates not classified in the above-mentioned databases we identified the protein peg.814 that has high similarity (87%) to the HIP57 protein from* Xenorhabdus budapestensis*, a protein homologous to the GroEL chaperone.* X. budapestensis* HIP57 caused* G. mellonella* larval bodies to blacken and die [38], a phenotype very similar to the phenotype observed for* A. faecalis* strain MOR02 in our experiments. It has been proposed that, in addition to the molecule chaperon role of GroEL, HIP57 could possess another novel function as a toxin insecticide [38]. Because of the results of the phenoloxidase (PO) activity analysis of* G. mellonella* larvae injected with HIP57* X. budapestensis*, the author suggested that HIP57 activates the PO cascade, which provides an extensive defense mechanism that is potentially responsible for* G. mellonella* larval death. The mode of action of this protein that has high similarity to GroEL and injectable toxicity to* G. mellonella* causes an increased phenoloxidase activity innate immune response that is stimulated by the injection [39]. Because different toxins have different activities against different pests, the proteases and toxins in* A. faecalis *MOR02 allow the exploration of novel options of pest control. It will be interesting in the future to mutate peg.814 and analyze its effects on* G. mellonella*.

In addition to the established associations of* Heterorhabditis* and* Steinernema* with* Photorhabdus* and* Xenorhabdus*, respectively, in this report, we describe the association between bacteria and nematodes. Although we found* A. faecalis *MOR02 associated with* S. feltiae, S. carpocapsae*, and* H. bacteriophora*, the function of these bacteria when they are associated with nematodes remains to be elucidated. It appears that the bacteria assist with the insect infection process, as well as with bacteria considered primary symbionts, as we demonstrated in the pathogenicity and toxicity assays. The reason that* A. faecalis* MOR02 is not able to establish a permanent symbiotic association with nematodes may be because it lacks the genes important for colonization. Easom et al. [40] analyzed mutants that had reduced ability to colonize the guts of infective juvenile (IJ) nematodes. They identified six different genetic loci (*pbgPE* operon,* galE*,* galU*,* asmA*,* hdfR*, and* proQ*) that were involved with the mutualistic colonization of IJ* H. bacteriophora *by* P. luminescens*. When we performed a search of these genes in the* A. faecalis *MOR02 genome we found two genes encoding proteins with 66% and 71% similarity to GalU (glucose-1-phosphate uridyltransferase or UDP-glucose pyrophosphorylase) and GalE (UDP-glucose 4-epimerase), respectively. The activities of these proteins are important for the production of polysaccharides, an important factor for colonization that is considered an important virulence factor in Gram-negative pathogens [40–43]. Therefore, it is likely that the presence of these two proteins enables the bacteria to colonize the gut of nematodes temporarily.

## 5. Conclusions 

In this work, we demonstrate pathogenicity and toxicity of bacteria isolated from the hemolymph of* G. mellonella* larvae identified as* A. faecalis* MOR02. These bacteria are pathogen to larvae insect and excrete toxic compounds and enzymes as chitinases that could be helping in the infection process. A possible bacteria-nematode association that is not inherited to progeny may be a strategy of dispersion that could be occurring in the environment.

Genomic analysis shows that these bacteria possess a wide repertoire of enzymes related to toxicity with a potential to be used as a biocontrol compound. We are now investigating the role of the protein peg.814 as a candidate in the toxicity of the bacteria.

## Supplementary Material

Figure S1: Graphical representation of a 5712 bp fragment from the contig1 showing the disposition of the transfers RNA genes tRNA-Val(gac) [121, 713-121, 789], tRNA-Leu(tag)[324, 903-324, 987], tRNA-Met(cat) [456, 223-456, 301] and tRNA Ala(tgc) [613, 447-613, 522], tRNA-Ile(gat) [613, 534-613, 610] in addition to the ribosomal genes 5S rRNA [609, 890-610, 001], 23S rRNA[610, 185-613, 067] and16SrRNA[613, 709-615, 233]
Figure S2: A phylogenetic analysis of one hundred and sixty five 16S rRNA sequences (available upon request) aligned with MUSCLE server version 3, calculating distances with the maximum likelihood method and inferring the evolutionary tree with the neighbor-joining method using 1000 replicates to calculate bootstrap values
Table S1: Functional analysis of the *Alcaligenes faecalis* Strain MOR02 proteome, comparing the genome with the annotation of the server RAST
Table S2: Analysis of the *Alcaligenes faecalis* strain MOR02 proteome in the Gene Ontology (GO) server
Table S3: Analysis of the *Alcaligenes faecalis* strain MOR02 proteome in the PFAM server.
Table S4: Analysis of the *Alcaligenes faecalis* strain MOR02 proteome in the KEGG Orthology (KO) server.
Table S5: Analysis of the *Alcaligenes faecalis* strain MOR02 proteome in the Clusters of Orthologous Groups of proteins (COG) server.

## Figures and Tables

**Table 1:** Mortality assessment in the pathogenicity assay of *A. faecalis* MOR02 and *E. coli* DH5*α* (carrying plasmid pT4-mCherry) on *G. mellonella* larvae.

Larvae treatment	Mortality (%)			
*A. faecalis* MOR02-Cherry	24 h after injection	48 h after injection	72 h after injection	>96 h after injection
Puncture	0	0	0	0
LB broth	0	0	0	0
240 CFU	0	0	3.33 ± 4.71	0
2,400 CFU	0	6.67 ± 4.71^a^	6.67 ± 9.43^a^	33.33 ± 9.42^a^
24,000 CFU	96.67 ± 4.71^a^	3.33 ± 4.71^a^	∗	∗
240,000 CFU	100^a^	∗	∗	∗

*E. coli *DH5*α*	24 h after injection	48 h after injection	72 h after injection	>96 h after injection

Puncture	0	10	0	0
LB broth	0	0	0	0
240 CFU	0	0	0	0
2,400 CFU	0	0	0	0
24,000 CFU	0	0	0	0
240,000 CFU	0	0	0	0

^a^Significant differences between values. Note: the percentage values shown are accumulative. ∗At this time all larvae were dead.

**Table 2:** Categorization of genes encoding proteins that were identified after an analysis on the RAST server and COG, GO, KO, and PFAM databases. All proteins are nonredundant.

Subgroups	Number genes encoding proteins
Host/environment interactions	
Host factor	2
Taxis	19
Flagellar	42
Signal transduction	34
Starvation	11
Metabolism of xenobiotics	9
Pathogenicity and toxicity	
Peptidases	54
Proteases	55
Lipases/esterases	88
Hemolysins	7
Invasins	2
Virulence	9
Toxins	9
Chitin deacetylase	1
Secretion system	29
Pathogenesis	7
Antibiotic synthesis	7
Drug/antibiotic resistance	
Drug/antibiotic transporter	68
Resistance	66
Beta-lactamase	20
Related to penicillin	12
Dihydrofolate reductase	3

## References

[1] Murfin K. E., Dillman A. R., Foster J. M. (2012). Nematode-bacterium symbioses-cooperation and conflict revealed in the ‘omics’ age. *Biological Bulletin*.

[2] Shapiro-Ilan D. I., Han R., Dolinksi C. (2012). Entomopathogenic nematode production and application technology. *Journal of Nematology*.

[3] Poinar G. O. (1979). *Nematodes for Biological Control of Insects*.

[4] Gouge D. H., Snyder J. L. (2006). Temporal association of entomopathogenic nematodes (Rhabditida: Steinernematidae and Heterorhabditidae) and bacteria. *Journal of Invertebrate Pathology*.

[5] Chaston J. M., Suen G., Tucker S. L. (2011). The entomopathogenic bacterial endosymbionts *Xenorhabdus* and *Photorhabdus*: convergent lifestyles from divergent genomes. *PLoS ONE*.

[6] Burnell A. M., Stock S. P. (2000). *Heterorhabditis, Steinernema* and their bacterial symbionts—lethal pathogens of insects. *Nematology*.

[7] Vaaje-Kolstad G., Horn S. J., Sørlie M., Eijsink V. G. H. (2013). The chitinolytic machinery of *Serratia marcescens*—a model system for enzymatic degradation of recalcitrant polysaccharides. *FEBS Journal*.

[8] Lysenko O., Weiser J. (1974). Bacteria associated with the nematode *Neoaplectana carpocapsae* and the pathogenicity of this complex for *Galleria mellonella* larvae. *Journal of Invertebrate Pathology*.

[9] Park H. W., Kim Y. O., Ha J.-S. (2011). Effects of associated bacteria on the pathogenicity and reproduction of the insect-parasitic nematode *Rhabditis blumi* (Nematoda: Rhabditida). *Canadian Journal of Microbiology*.

[10] Meyer F., Paarmann D., D'Souza M. (2008). The metagenomics RAST server—a public resource for the automatic phylogenetic and functional analysis of metagenomes. *BMC Bioinformatics*.

[11] Lagesen K., Hallin P., Rødland E. A., Stærfeldt H.-H., Rognes T., Ussery D. W. (2007). RNAmmer: consistent and rapid annotation of ribosomal RNA genes. *Nucleic Acids Research*.

[12] Schattner P., Brooks A. N., Lowe T. M. (2005). The tRNAscan-SE, snoscan and snoGPS web servers for the detection of tRNAs and snoRNAs. *Nucleic Acids Research*.

[13] Laslett D., Canback B. (2004). ARAGORN, a program to detect tRNA genes and tmRNA genes in nucleotide sequences. *Nucleic Acids Research*.

[14] Tatusov R. L., Fedorova N. D., Jackson J. D. (2003). The COG database: an updated vesion includes eukaryotes. *BMC Bioinformatics*.

[15] Raman M., Banu S. S., Gomathinayagam S., Raj G. D. (2011). Lesion scoring technique for assesing the virulence and pathogenicity of Indian field isolates of avian *Eimeria* species. *Veterinarski Arhiv*.

[16] Ashburner M., Ball C. A., Blake J. A. (2000). Gene Ontology: tool for the unification of biology. *Nature Genetics*.

[17] Khalafalla R. E., Müller U., Shahiduzzaman M. (2011). Effects of curcumin (diferuloylmethane) on *Eimeria tenella* sporozoites in vitro. *Parasitology Research*.

[18] Ogata H., Goto S., Sato K., Fujibuchi W., Bono H., Kanehisa M. (1999). KEGG: Kyoto encyclopedia of genes and genomes. *Nucleic Acids Research*.

[19] Schares G., Pantchev N., Barutzki D., Heydorn A. O., Bauer C., Conraths F. J. (2005). Oocysts of *Neospora caninum*, *Hammondia heydorni*, *Toxoplasma gondii* and *Hammondia hammondi* in faeces collected from dogs in Germany. *International Journal for Parasitology*.

[20] Edgar R. C. (2004). MUSCLE: a multiple sequence alignment method with reduced time and space complexity. *BMC Bioinformatics*.

[21] Tamura K., Stecher G., Peterson D., Filipski A., Kumar S. (2013). MEGA6: molecular evolutionary genetics analysis version 6.0. *Molecular Biology and Evolution*.

[22] Giammanco G. M., Grimont P. A. D., Grimont F., Lefevre M., Pignato S. (2011). Phylogenetic analysis of the genera *Proteus*, *Morganella* and *Providencia* by comparison of rpoB gene sequences of type and clinical strains suggests the reclassification of *Proteus myxofaciens* in a new genus, *Cosenzaea* gen. nov., as *Cosenzaea myxofaciens* comb. nov. *International Journal of Systematic and Evolutionary Microbiology*.

[23] Wood T. K., González Barrios A. F., Herzberg M., Lee J. (2006). Motility influences biofilm architecture in *Escherichia coli*. *Applied Microbiology and Biotechnology*.

[24] Kilburn J. O., O'Donnell K. F., Silcox V. A., David H. L. (1973). Preparation of a stable mycobacterial tween hydrolysis test substrate. *Journal of Applied Microbiology*.

[25] Aylward F. O., Tremmel D. M., Starrett G. J. (2013). Complete genome of *Serratia* sp. strain FGI 94, a strain associated with leaf-cutter ant fungus gardens. *Genome Announcements*.

[26] Hernández-Mendoza A., Lozano-Aguirre Beltrán LF., Martínez-Ocampo F., Quiroz-Castañeda RE., Dantán-González E. (2014). A newly sequenced *Alcaligenes faecalis* strain: implications for novel temporal symbiotic relationships. *Genome Announcements*.

[27] Laslett D., Canback B. (2004). ARAGORN, a program to detect tRNA genes and tmRNA genes in nucleotide sequences. *Nucleic Acids Research*.

[28] Annamalai N., Rajeswari M. V., Vijayalakshmi S., Balasubramanian T. (2011). Purification and characterization of chitinase from *Alcaligenes faecalis* AU02 by utilizing marine wastes and its antioxidant activity. *Annals of Microbiology*.

[29] Garrity G., Brenner D. J., Staley J. T. (2006). *Bergey's Manual of Systematic Bacteriology: Volume Two: The Proteobacteria (Part C)*.

[30] Hunt D. E., Gevers D., Vahora N. M., Polz M. F. (2008). Conservation of the chitin utilization pathway in the Vibrionaceae. *Applied and Environmental Microbiology*.

[31] Zhao Y., Park R. D., Muzzarelli R. A. A. (2010). Chitin deacetylases: properties and applications. *Marine Drugs*.

[32] Kaur K., Dattajirao V., Shrivastava V., Bhardwaj U. (2012). Isolation and characterization of chitosan-producing bacteria from beaches of Chennai, India. *Enzyme Research*.

[33] Mahlen S. D. (2011). *Serratia* infections: from military experiments to current practice. *Clinical Microbiology Reviews*.

[34] Shanks R. M. Q., Lahr R. M., Stella N. A. (2013). A *Serratia marcescens* PigP homolog controls prodigiosin biosynthesis, swarming motility and hemolysis and is regulated by cAMP-CRP and HexS. *PLoS ONE*.

[35] Phung L. T., Trimble W. L., Meyer F., Gilbert J. A., Silver S. (2012). Draft genome sequence of *Alcaligenes faecalis* subsp. *faecalis* NCIB 8687 (CCUG 2071). *Journal of Bacteriology*.

[36] Sandkvist M. (2001). Type II secretion and pathogenesis. *Infection and Immunity*.

[37] Nivaskumar M., Francetic O. (2014). Type II secretion system: a magic beanstalk or a protein escalator. *Biochimica et Biophysica Acta—Molecular Cell Research*.

[38] Yang J., Zeng H. M., Lin H. F. (2012). An insecticidal protein from *Xenorhabdus budapestensis* that results in prophenoloxidase activation in the wax moth, *Galleria mellonella*. *Journal of Invertebrate Pathology*.

[39] Castagnola A., Stock S. P. (2014). Common virulence factors and tissue targets of entomopathogenic bacteria for biological control of lepidopteran pests. *Insects*.

[40] Easom C. A., Joyce S. A., Clarke D. J. (2010). Identification of genes involved in the mutualistic colonization of the nematode *Heterorhabditis bacteriophora* by the bacterium *Photorhabdus luminescens*. *BMC Microbiology*.

[41] Zou Y., Feng S., Xu C. (2013). The role of galU and galE of *Haemophilus parasuis* SC096 in serum resistance and biofilm formation. *Veterinary Microbiology*.

[42] Jiang S.-S., Lin T.-Y., Wang W.-B., Liu M.-C., Hsueh P.-R., Liaw S.-J. (2010). Characterization of UDP-glucose dehydrogenase and UDP-glucose pyrophosphorylase mutants of *Proteus mirabilis*: defectiveness in polymyxin B resistance, swarming, and virulence. *Antimicrobial Agents and Chemotherapy*.

[43] Ramjeet M., Cox A. D., Hancock M. A. (2008). Mutation in the LPS outer core biosynthesis gene, *galU*, affects LPS interaction with the RTX toxins ApxI and ApxII and cytolytic activity of *Actinobacillus pleuropneumoniae* serotype 1. *Molecular Microbiology*.

