# Supplementary figures and images for: Identification of a New Alcaligenes faecalis Strain MOR02 and Assessment of Its Toxicity and Pathogenicity to Insects
Source: Biomed Res Int. 2015 Jan 18;2015:570243. doi: 10.1155/2015/570243 (PMC4312618; doi:10.1155/2015/570243)

# NODE\_26\_length\_5712\_cov\_454.389008 contig

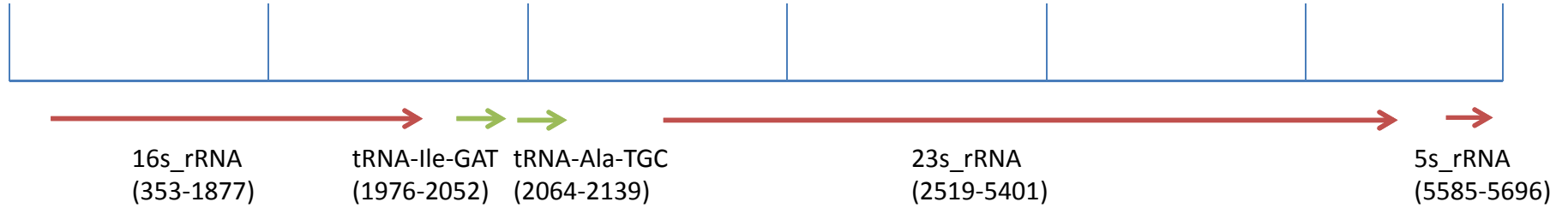

5712 pb

Supplement: Supplementary file 1 — Figure S1: Graphical representation of a 5712 bp fragment from the contig1 showing the disposition of the transfers RNA genes tRNA-Val(gac) [121, 713-121, 789], tRNA-Leu(tag)[324, 903-324, 987], tRNA-Met(cat) [456, 223-456, 301] and tRNA Ala(tgc) [613, 447-613, 522], tRNA-Ile(gat) [613, 534-613, 610] in addition to the ribosomal genes 5S rRNA [609, 890-610, 001], 23S rRNA[610, 185-613, 067] and16SrRNA[613, 709-615, 233] Figure S2: A phylogenetic analysis of one hundred and sixty five 16S rRNA sequences (available upon request) aligned with MUSCLE server version 3, calculating distances with the maximum likelihood method and inferring the evolutionary tree with the neighbor-joining method using 1000 replicates to calculate bootstrap values Table S1: Functional analysis of the Alcaligenes faecalis Strain MOR02 proteome, comparing the genome with the annotation of the server RAST Table S2: Analysis of the Alcaligenes faecalis strain MOR02 proteome in the Gene Ontology (GO) server Table S3: Analysis of the Alcaligenes faecalis strain MOR02 proteome in the PFAM server. Table S4: Analysis of the Alcaligenes faecalis strain MOR02 proteome in the KEGG Orthology (KO) server. Table S5: Analysis of the Alcaligenes faecalis strain MOR02 proteome in the Clusters of Orthologous Groups of proteins (COG) server. [file 570243.f1.zip › Supplementary materials/FiguraS1.pdf]

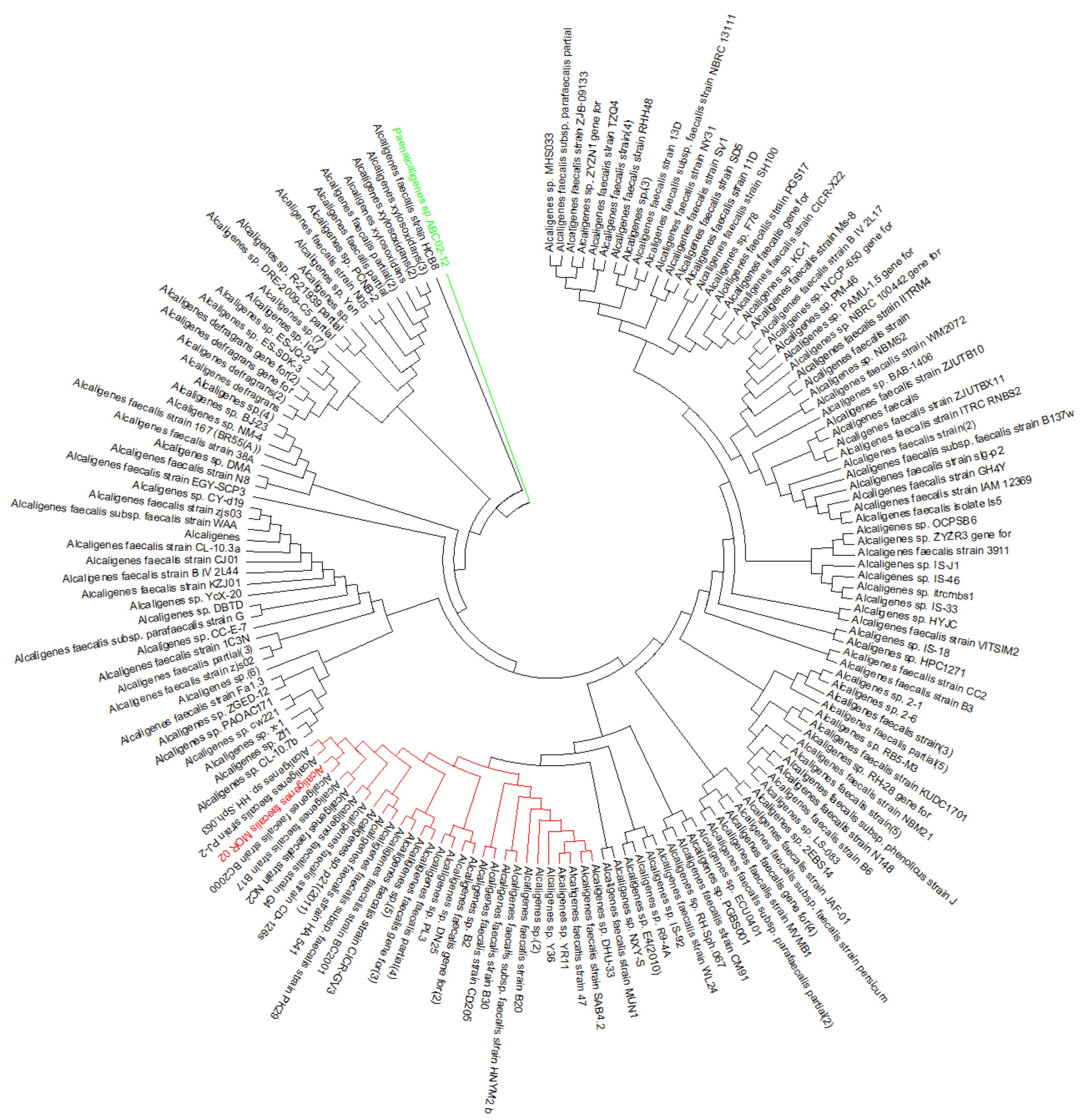

Supplement: Supplementary file 1 — Figure S1: Graphical representation of a 5712 bp fragment from the contig1 showing the disposition of the transfers RNA genes tRNA-Val(gac) [121, 713-121, 789], tRNA-Leu(tag)[324, 903-324, 987], tRNA-Met(cat) [456, 223-456, 301] and tRNA Ala(tgc) [613, 447-613, 522], tRNA-Ile(gat) [613, 534-613, 610] in addition to the ribosomal genes 5S rRNA [609, 890-610, 001], 23S rRNA[610, 185-613, 067] and16SrRNA[613, 709-615, 233] Figure S2: A phylogenetic analysis of one hundred and sixty five 16S rRNA sequences (available upon request) aligned with MUSCLE server version 3, calculating distances with the maximum likelihood method and inferring the evolutionary tree with the neighbor-joining method using 1000 replicates to calculate bootstrap values Table S1: Functional analysis of the Alcaligenes faecalis Strain MOR02 proteome, comparing the genome with the annotation of the server RAST Table S2: Analysis of the Alcaligenes faecalis strain MOR02 proteome in the Gene Ontology (GO) server Table S3: Analysis of the Alcaligenes faecalis strain MOR02 proteome in the PFAM server. Table S4: Analysis of the Alcaligenes faecalis strain MOR02 proteome in the KEGG Orthology (KO) server. Table S5: Analysis of the Alcaligenes faecalis strain MOR02 proteome in the Clusters of Orthologous Groups of proteins (COG) server. [file 570243.f1.zip › Supplementary materials/FiguraS2.pdf]
